# PReS-FINAL-2046: Uveitis in the Nordic juvenile idiopathic arthritis cohort; high incidence, frequent complications, and gender-associated risk factors

**DOI:** 10.1186/1546-0096-11-S2-P59

**Published:** 2013-12-05

**Authors:** EB Nordal, L Berntson, K Aalto, S Peltoniemi, S Nielsen, T Herlin, A Fasth, M Rygg, the Nordic Study Group of Pediatric Rheumatology (NoSPeR)

**Affiliations:** 1 Department of Pediatrics, University Hospital of North Norway, Norway; 2 Deaprtment of Clinical Medicine, University of Tromsø, Tromsø, Norway; 3 Department of Pediatrics, Uppsala University Children's Hospital, Uppsala, Sweden; 4 Department of Pediatrics, Helsinki Children's University Hospital, Helsinki, Finland; 5 Department of Pediatric Rheumatology, Rigshospitalet University Hospital, Copenhagen, Denmark; 6 Department of Pediatrics, Århus University Hospital, Århus, Denmark; 7 Department of Pediatrics, University of Gothenburg, Gothenburg, Sweden; 8 Department of Laboratory Medicine, Children's and Women's Health, Norwegian University of Science and Technology, Norway; 9 Department of Pediatrics, St Olav University Hospital, Trondheim, Norway

## Introduction

Uveitis is the most common extra-articular manifestation of juvenile idiopathic arthritis (JIA). Both incidence and reported outcome varies, and population-based data are scarce. Early identification of asymptomatic cases is important in order to avoid complications and reduced vision.

## Objectives

The aim of the study was to identify incidence, risk factors and outcome of uveitis in a cohort of Nordic children with JIA followed for eight years in a population-based setting.

## Methods

Consecutive cases of JIA from defined geographical areas of Denmark, Finland, Sweden and Norway with disease onset in 1997-2000 were included. The study aimed to be as close to population-based as possible, as centers participated only if they were able to include all children diagnosed with JIA in their catchment area. Clinical and ophthalmologic data were registered at regular follow-up visits for 7-12 years after disease onset.

## Results

Of 500 children included at baseline, 440 were followed for at least 7 years, and 389 (78%) had available ophtalmologic data. Uveitis developed in 89 (23%) of the 389 children; 59 girls and 30 boys, acute uveitis in 12 and chronic uveitis in 77 children. Fifty percent (6/12) of patients with acute uveitis were HLA-B27 positive. Young age at onset of JIA was a significant predictor of chronic uveitis in girls (p = 0.0001), but not in boys (p = 0.47). Also the presence of anti-nuclear antibodies (ANA Hep-2) (p = 0.003) was a significant predictor in girls (p = 0.003), but not in boys (p = 0.05). Neither female gender nor oligoarticular onset JIA category was significantly associated with uveitis. Chronic uveitis was diagnosed at a median of 0.8 years after onset of disease, and in 88% within the first four years after onset of disease. The longest interval between JIA onset and uveitis development was 8.6 years. Complications occurred in 39 eyes in 22 of the 89 patients with uveitis (25%), glaucoma occurred in 19 eyes and cataract in 14 eyes. At the last visit visus was <0.5 in 10 eyes in 9 patients.

## Conclusion

We found high incidence of uveitis among Nordic children with JIA, and the majority develop early after onset of disease. Age at onset of JIA and presence of ANA Hep-2 were associated with development of uveitis in girls, but not in boys. Complications were present seven years after onset of arthritis in 25%, showing that uveitis contributes significantly to morbidity and disability in children with JIA.

## Disclosure of interest

None declared.

